# CIP2A down regulation enhances the sensitivity of pancreatic cancer cells to gemcitabine

**DOI:** 10.18632/oncotarget.7447

**Published:** 2016-02-17

**Authors:** Peng Xu, Jie Yao, Jin He, Long Zhao, Xiaodong Wang, Zhennan Li, Jianjun Qian

**Affiliations:** ^1^ Department of Hepatobiliary and Pancreatic Surgery, Northern Jiangsu People's Hospital, Clinical medical college of Yangzhou University, Yangzhou city, Jiangsu Provence of P. R. China, 225001

**Keywords:** CIP2A, pancreatic ductal adenocarcinoma, gemcitabine, prognosis, overall survival

## Abstract

Cancerous inhibitor of protein phosphatase 2A (CIP2A) is an oncoprotein which participates in inhibiting tumor apoptosis in pancreatic cancer cells. Using immunohistochemical staining, we investigated the expression of CIP2A protein in 72 cases of human pancreatic ductal adenocarcinoma (PDAC) tissue and 27 cases of adjacent normal pancreatic tissue. The positive rate of CIP2A protein expression in pancreatic cancer tissue was70.83 %, which was significantly higher than that in adjacent non- cancerous pancreatic tissue (11.11%). The expression of CIP2A was found to be correlated with TNM stage, but not correlated with age, gender, tumor location, smoking status, alcohol consumption, diabetes, high blood pressure, BMI, tumor size, lymph node metastasis or distant metastases. Kaplan- Meier survival analysis showed that patients with positive CIP2A protein expression had a lower overall survival rate than patients without CIP2A expression. COX regression analysis indicated that expression of CIP2A was an independent prognostic factor for pancreatic ductal adenocarcinoma. In addition, down-regulation of CIP2A inhibited cell proliferation and increased sensitivity to gemcitabine in pancreatic cancer cells by decreasing AKT signaling pathway. Our results indicated that down-regulation of CIP2A could be a novel therapeutic strategy for pancreatic cancer.

## INTRODUCTION

Pancreatic cancer is one of the most devastating human malignancies. In recent years, the incidence of pancreatic cancer grows rapidly. Pancreatic cancer is difficult to diagnose at early stage and is often found to metastasize to distant organs, because there is no specific symptoms at the beginning of the disease [1, 2]. Although many treatment modalities, for example, surgery, radiotherapy and chemotherapy, are used to treat pancreatic cancer, the median overall survival was only approximately 27 months in patients with resectable disease [3]. The aggressiveness of pancreatic cancer was partially due to its intrinsic and extrinsic drug resistance to chemotherapy [4]. The standard chemotherapy for advanced pancreatic cancer patients was gemcitabine or combined with other chemotherapeutic drugs [5, 6]. It is necessary to understand the molecular mechanism of drug resistance to gemcitabine in pancreatic cancer, particularly in pancreatic ductal adenocarcinoma, which is the most common type of pancreatic cancer.

Tumor progression is linked to genetic alterations. Recent studies have found that Cancerous inhibitor of protein phosphatase 2A (CIP2A) is expressed in pancreatic cancer [7,8], which play a role in cancer as an oncoprotein. CIP2A gene is located in the chromosome 3 at 3q13-q13.2 [9]. An increased expression of CIP2A protein was found in cholangiocarcinoma, gastric cancer, breast cancer, prostate cancer, lung cancer and in head and neck squamous cell carcinomas [10–15].

In this study, we evaluated the expression of CIP2A and its relationship to the prognosis and drug resistance in pancreatic ductal adenocarcinoma. Furthermore, the effect of CIP2A in cancer cell proliferation and apoptosis was investigated in pancreatic cancer cells. The association of CIP2A expression with p-AKT level was analyzed in drug resistant pancreatic cancer cells.

## RESULTS

### Differential expression of CIP2A in pancreatic cancer and adjacent normal tissue

**Figure 1:**
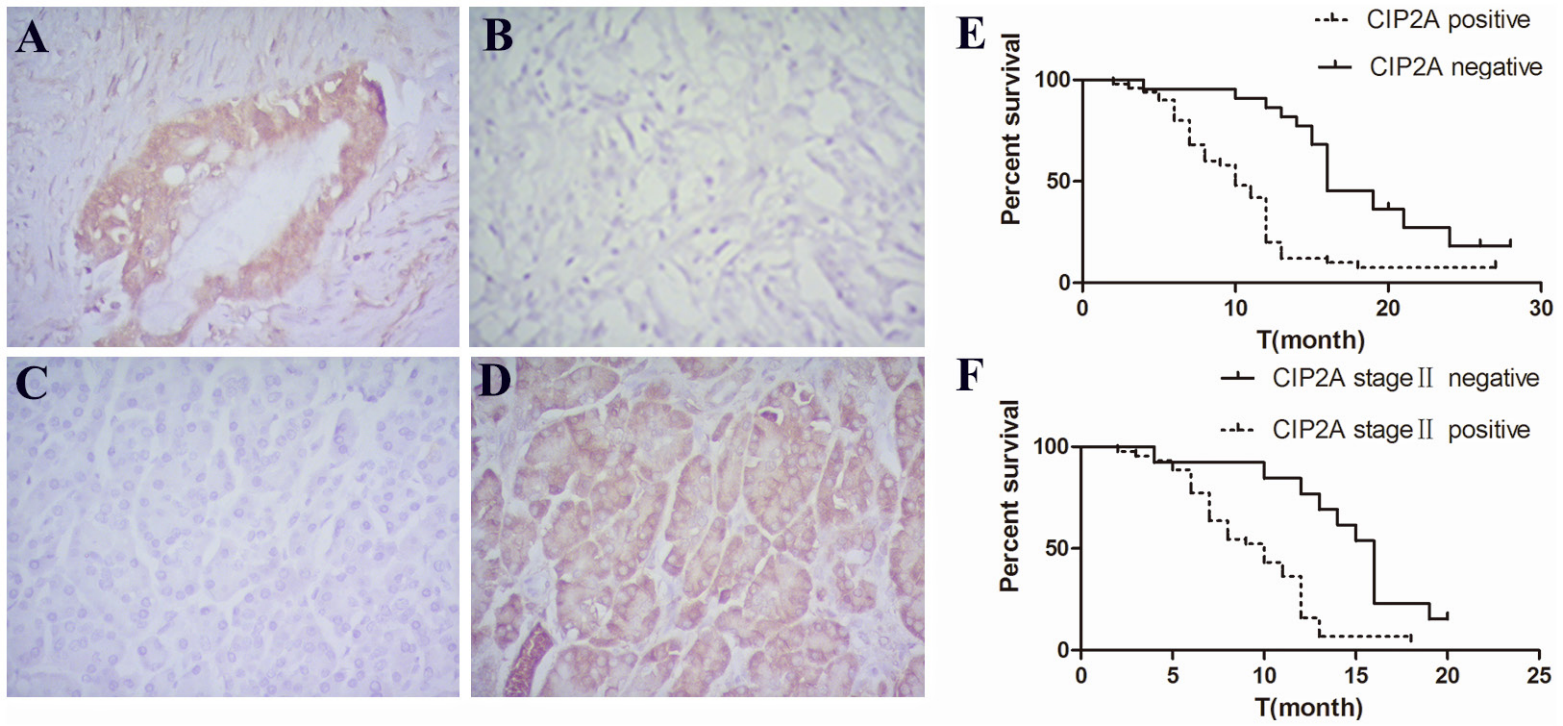
Expression of CIP2A in pancreatic cancer and adjacent normal pancreatic tissues and its correlation with overall survival in patients **A.** Positive expression of CIP2A in pancreatic cancer tissue(X400). **B.** Negative expression of CIP2A in pancreatic cancer tissue(X400). **C.** Negative expression of CIP2A in adjacent normal pancreatic tissue(X400). **D.** Positive expression of CIP2A in adjacent normal pancreatic tissue(X400) **E.** Correlation of CIP2A expression and overall survival in all patients. **F.** Correlation of CIP2A expression and overall survival in stage II patients.

To investigate if there is a difference in the expression of CIP2A protein between pancreatic cancer and normal tissues, expression of CIP2A was detected using immunohistochemistry. We found 51 out of 72 (70.83%) cancer cases had positive CIP2A expression in the cytoplasm of pancreatic cancer cells (Figure 1A and 1B). In the adjacent normal pancreatic tissue, only 3 out of 27 (11.11%) cases were positive for CIP2A expression (Figure 1C and 1D). The difference of expression between PDAC tissue and adjacent normal pancreatic tissues was significant (*p*<0.05) (Table 1). This result indicated that pancreatic cancer over-expressed CIP2A similar to cancers in other organs.

**Table 1:** Expression of CIP2A in PDAC and adjacent normal pancreatic tissue [n, n(%)]

Group	CIP2A			
	Positive	Negative	χ^2^ value	P value
PDAC tissue	51(70.83%)	21(29.17%)	28.249	0.000
Normal pancreatic tissue	3(11.11%)	24(88.89%)		

### Correlation between the expression of CIP2A and patients' general characteristics

The expression of CIP2A protein was examined in the 72 PDAC patients based on patients' general characteristics, such as age, gender, tumor location, TNM stage et al. The detail of the parameters examined was listed in Table 2. There was no significant difference between the CIP2A expression and age, gender, tumor location, smoking status, alcohol drinking, diabetes, high blood pressure, BMI and tumor size (*p*>0.05). However, the expression of CIP2A was positively correlated with TNM stage (*p*<0.05) (Table 2).

**Table 2:** CIP2A protein expression and clinicopathologic features in PDAC patients

Characteristics	N	CIP2A Positive	χ^2^	*P*
Age(year)			2.268	0.132
<60	23	19		
≥60	49	32		
Gender			1.4	0.237
Male	42	32		
Female	30	19		
Tumor location			0.261	0.610
Head	61	42		
Body or Tail	11	9		
Smoking index^a^				
Yes	35	25	0.012	0.914
No	37	26		
Alcohol^b^			0.385	0.535
Yes	28	21		
No	44	30		
Diabetes			1.008	0.315
Yes	27	21		
No	45	30		
High blood pressure			0.758	0.384
Yes	13	11		
no	59	40		
BMI			3.178	0.075
18.5≤BMI<24	51	33		
BMI < 18.5,24≤BMI,	21	18		
Lymph node metastasis			3.698	0.054
N0	50	32		
N1	22	19		
Distant metastases			0.017	0.859
Yes	2	2		
No	70	49		
TNM stage			6.936	0.008
I	15	6		
II+III+IV	57	45		
Tumor size			0.102	0.75
>2cm	19	14		
≤4cm	53	37		

a Smoking at least one pack a day for more than a year

b Drinking is defined as at least 1 drink per week for at least 1 year

### Correlation between the expression of CIP2A protein and clinicopathological characteristics

The follow up of our study was ended in Jun 2014. The median overall survival time was 12 months. 47 out of 72 patients died between 2 and 28 months. Cumulative survival was analyzed among the patients with PDAC by Log rank test. The result indicated that there was a significant difference in the overall survival in the patients with high and low expression of CIP2A protein (log rank=14.732, *p*=0.000) (Figure 1E). Among these patients, 55 cases of the patients have underwent surgery in stage II. Our results suggested that patients in stage II with low CIP2A expression had a better prognosis and longer overall survival than those with high CIP2A expression (log rank=9.841, *p*=0.002) (Figure 1F). A multivariate progression analysis by Cox regression showed that CIP2A expression was a significant independent factor in PDAC patients (Hazard ratio(*HR) =2.268, p=*0.008).

### Pancreatic cancer cells over-expressed CIP2A and knockdown of CIP2A decreased CIP2A expression

**Figure 2:**
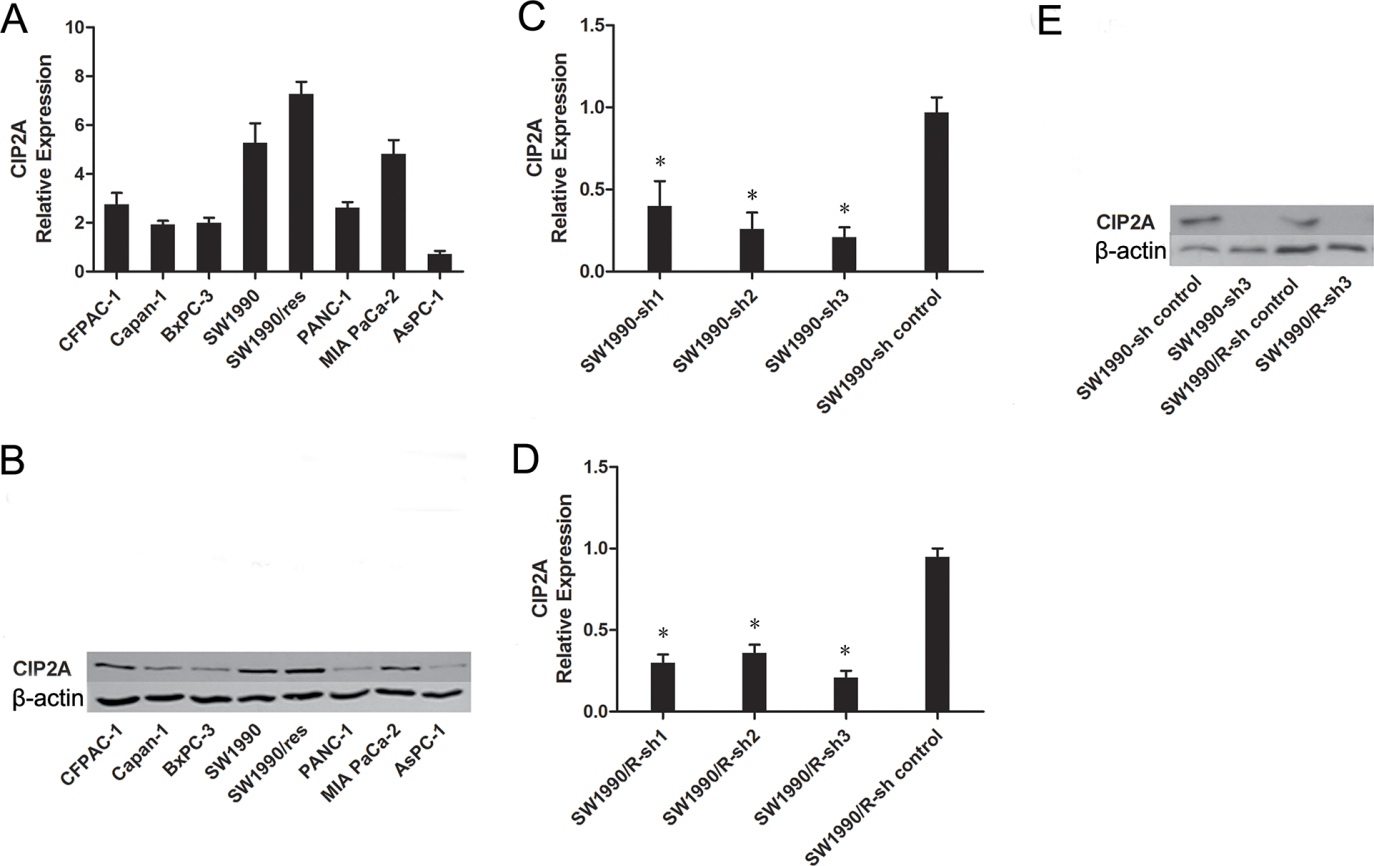
Expression of CIP2A in pancreatic cancer cell lines **A.** Relative CIP2A mRNA level in pancreatic cancer cell lines. **B.** Expression of CIP2A protein in pancreatic cancer cell lines. **C.** The mRNA levels of CIP2A in SW1990 cells after CIP2A-sh transfection. **D.** The mRNA levels of CIP2A in SW1990/R cells after CIP2A-sh transfection **E.** Western blot detection of CIP2A protein in pancreatic cancer cell lines after CIP2A-sh transfection. (* = *p* ≤ 0.05).

The above results promoted us to investigate if CIP2A are over-expressed in pancreatic cancer cells. We tested CIP2A mRNA expression in 9 pancreatic cancer cell lines. To quantitate CIP2A mRNA level of cell lines, total cDNAs of hTERT-immortalized pancreatic ductal epithelial cells (DT) were used as a non-transformed control. Compared to the DT cell, CIP2A mRNA expression was high in 8 out of the 9 (88%) pancreatic cancer cell lines (Figure 2A). Western blots analysis confirmed that the protein level of CIP2A increased in pancreatic cancer cells (Figure 2B).

After knockdown of CIP2A, qRT–PCR analysis showed that the mRNA of CIP2A knockdown SW1990 cells had an 80% lower expression when compared with control groups (*p*<0.05), while the mRNA of CIP2A knockdown SW1990/R cells had an 75% lower expression when compared with control groups (*p*<0.05) (Figure 2C and 2D). Western blots analysis confirmed these results (Figure 2E).

### Knock-down CIP2A increased sensitivity to gemcitabine in pancreatic cancer cells

**Figure 3:**
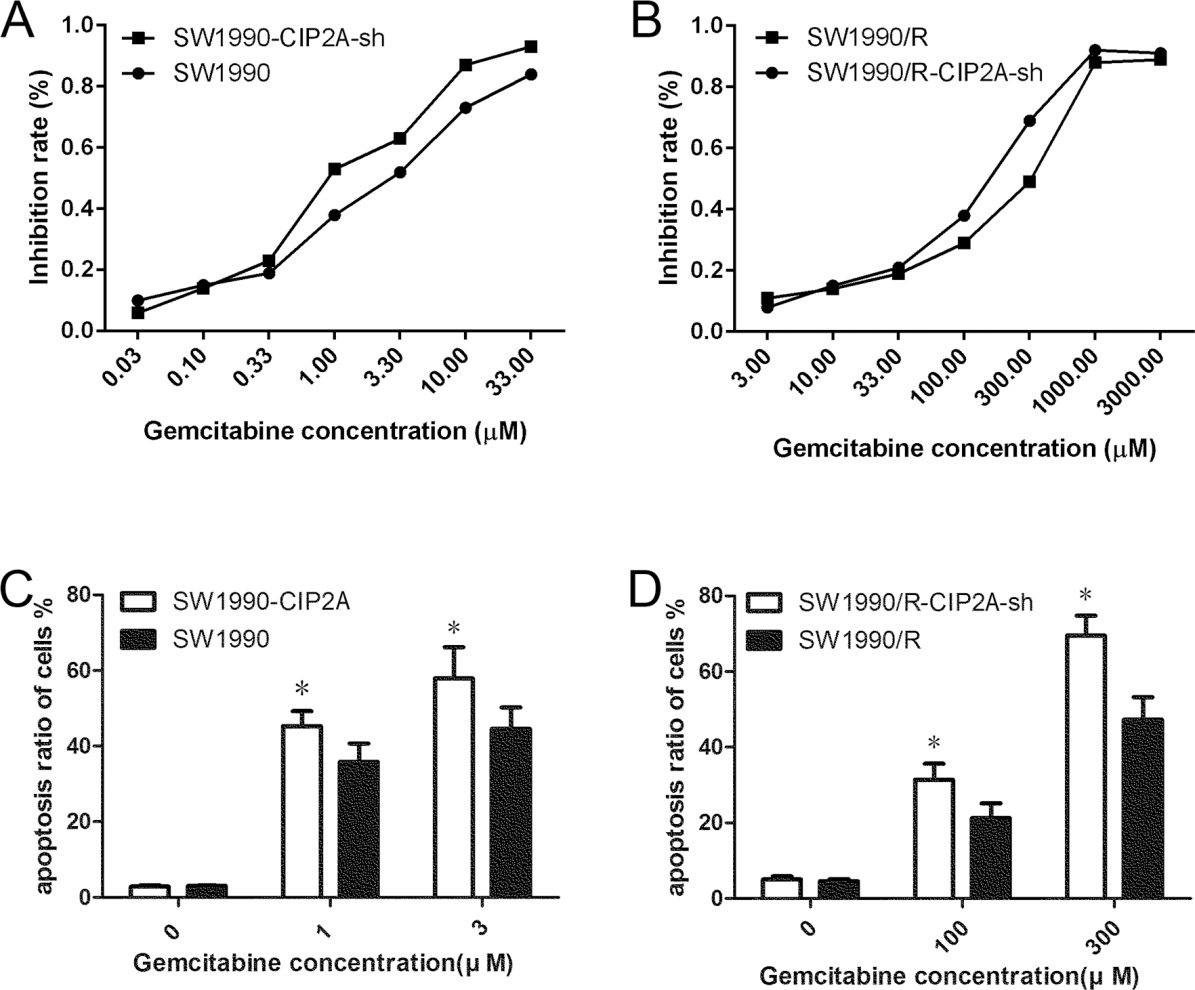
Knock-down CIP2A increased sensitivity to gemcitabine in PDAC cells IC50 decreased after CIP2A knock-down in both SW1990 **A.** and SW1990/R **B.** cells. **C.** Flow cytometric analysis using PI and Annexin V staining after gemcitabine treatments at different concentrations in SW1990 and SW1990-CIP2A cells. **D.** Flow cytometric analysis after gemcitabine treatments at different concentrations in SW1990/R and SW1990/R-CIP2A cells (* = *p* ≤ 0.05).

Since CIP2A are over-expressed in pancreatic cells, we asked whether there is a relation between CIP2A expression and gemcitabine sensitivity. To explore this possibility, cells before and after CIP2A knockdown were treated with different concentrations of gemcitabine. Inhibition rates and the half maximal inhibitory concentration (IC50) were investigated. We found that both the CIP2A knockdown SW1990 cells and SW1990/R cells demonstrated lower IC50 than SW1990 and SW1990/R (23.59±9.30 vs. 4.38±1.59; n=3, *p*=0.024), suggesting that cells with CIP2A knockdown were more sensitive to gemcitabine treatment (Figure 3A and 3B).

To further explore if CIP2A knockdown affected cell proliferation or apoptosis, we performed flow cytometric analysis using PI and Annexin V staining following gemcitabine treatments. Compared to SW1990 cells, SW1990-CIP2A-sh cells showed a high sensitivity to gemcitabine. SW1990-CIP2A cells treated with 0, 1, and 3μM gemcitabine showed more apoptotic cells than that in SW1990 cells receiving the same treatment (2.82±0.41 vs.3.01±0.06%,45.31±3.89 vs. 35.81±4.88%, 57.90±8.29 vs. 44.60±5.65%, respectively; Student's *t*-test, n=3, *p*<0.05, Figure 3C). Similarly, SW1990/R and SW1990/R-CIP2A-sh cells were treated with 0, 100 and 300 μM gemcitabine, respectively. SW1990-CIP2A-sh cells also showed more apoptotic cells than that in SW1990/R cells (4.54±0.59 vs. 5.03±0.81%, 31.43±4.23 vs.21.23±3.95%, 69.53±5.23 vs. 47.32±5.93%, respectively. Student's *t*-test, n=3, *p*<0.05, Figure 3D). These results indicated that down regulation of CIP2A can induced cell apoptosis, suggesting that over-expression of CIP2A could be one of the factors contributed to cancer cells resistant to gemcitabine treatment.

### Knock-down CIP2A down regulated p-AKT and BLC-2 in pancreatic cancer cells

**Figure 4:**
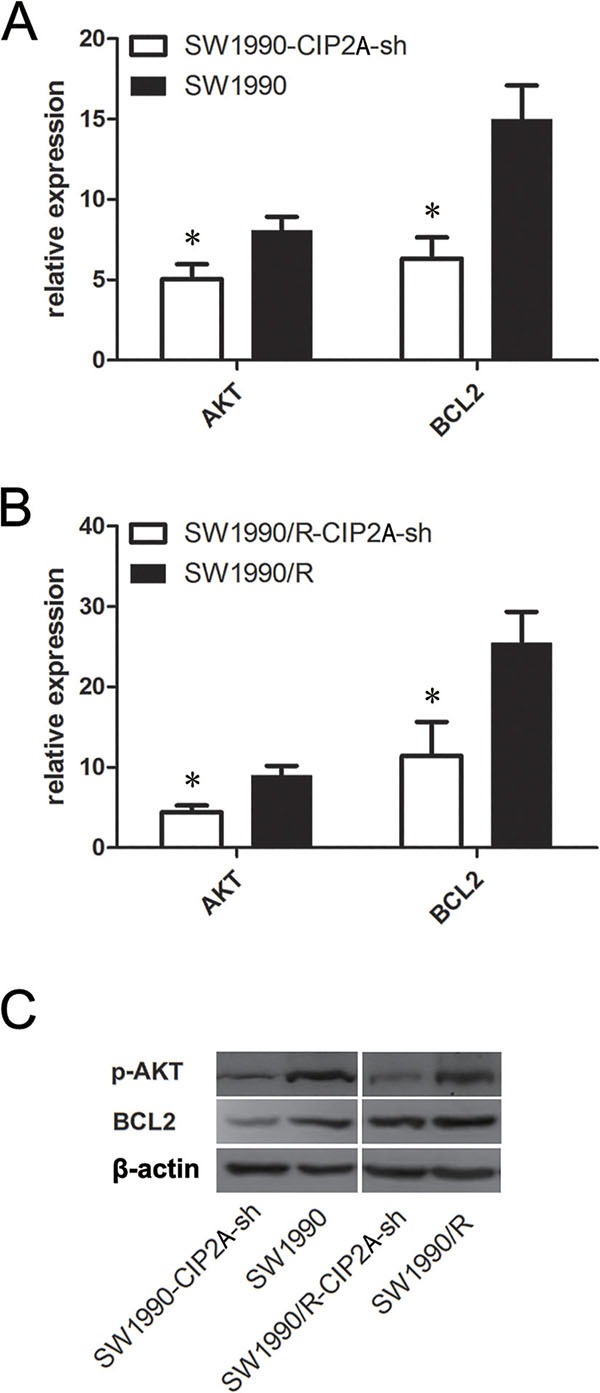
Knock-down CIP2A decreased p-AKT and BLC2 in pancreatic cancer cells Knockdown of CIP2A decreased AKT and BCL2 at mRNA level in both SW1990 **A.** and SW1990/R **B.** cells. Western blot **C.** showed decreased protein level of p-AKT and BLC2. (* = *p* ≤ 0.05).

To elucidate the underlying mechanisms by which CIP2A promoted chemo-resistance of pancreatic cancer cells, we investigated the expression of several key proteins in CIP2A knockdown SW1990 and SW1990/R cells. Knockdown of CIP2A resulted in decreased mRNA levels of AKT and BCL2 in both SW1990 and SW1990/R cells (Figure 4A and 4B). Similar changes were observed in p-AKT and BCL2 protein levels (Figure 4C). These data showed that knocking down CIP2A inhibited cell proliferation and increased sensitivity to gemcitabine in pancreatic cancer cells by decreasing AKT signaling pathway.

## DISCUSSIONS

In this study, we applied immunohistochemical staining to investigate the expression of CIP2A protein in normal and pancreatic tumor tissues. We found over-expression of CIP2A protein in PDAC tissues. The over-expression of CIP2A protein has been previously reported in a variety of cancer types [12–19]. This result supported that CIP2A gene may be a candidate oncogene for many tumor types. The over-expression of CIP2A had no correlation with age, gender, tumor location, smoking status, alcohol consumption, diabetes, high blood pressure, BMI, distant metastases, tumor size and lymph node metastasis. However, it correlated with TNM stage. Our study indicated that over-expression of CIP2A was related to the progression of PDAC. There was a report that CIP2A protein can promote proliferation of gastric cancer cell and the inhibition of CIP2A makes the tumor cells undergo senescence [20]. It was proposed that CIP2A protein promotes cell proliferation by regulating MYC-mediated gene expression [21], and it mediates cancer progression through interacting with the AKT-mTOR signaling pathway [22, 23].

Our clinicopathological analysis showed that expression of CIP2A was negatively correlated with the patients' overall survival. The negative expression of CIP2A protein was related to good prognosis in PDAC. Our COX regression analysis showed that CIP2A expression was a significant independent factor for PDAC.

Evidence has suggested that CIP2A was associated with proliferation and apoptosis of tumor [12–19]. However, evidence was lacking in concern with the relationship between CIP2A and chemotherapeutic treatments. We explore the relationship concerning the level of CIP2A and drug sensitivity. We knocked down CIP2A in pancreatic cancer cells and investigated the drug sensitivity of cancer cells to gemcitabine treatment. The result showed that knocking down CIP2A increased sensitivity to gemcitabine in pancreatic cancer cells. We further explored the signaling pathway related CIP2A biological functions. Since p-AKT is a key protein in PI3A-AKT signaling pathways [24], we investigated its relation with CIP2A expression. We found that down regulating CIP2A resulted in the down regulation of p-AKT gene in both pancreatic cancer cells and drug-resistant cells. We suggested that the interaction between these two genes may be the mechanism that determines the biological behavior of pancreatic cancer cells. Such interaction might involve in drug resistance of pancreatic cancer.

Taken together, we show that CIP2A is over-expressed in PDAC and could be an oncoprotein, CIP2A can inhibit apoptosis of pancreatic cancer cells. The biological function of CIP2A in stimulating tumor progression and drug resistance may relate to AKT signaling pathway. CIP2A could be a prognostic biomarker and a biomarker for molecular targeting therapy.

## MATERIALS AND METHODS

### Patients and tissue specimen

This study was approved by the institutional review board of Northern Jiangsu People's Hospital, Yang Zhou, China. Written informed consent was obtained before tissue acquisition according to ethical guideline. 72 pancreatic cancer tissues and 27 adjacent noncancerous pancreatic tissues from patients with pancreatic ductal adenocarcinoma, who underwent surgical resection at the department of Hepatobiliary and Pancreatic Surgery Northern Jiangsu people's hospital between January 2010 and December 2012 were collected. All the patients had not received radiotherapy or chemotherapy before surgery. The patient population consisted of 42 men and 30 women with a median age of 63 years old (aged from 34 to 81 years old). 61 cases of tumors were located in the head,11 cases of tumors were located in the body or tail. All the tissue specimens were independently evaluated by two pathologists without knowing the characteristics of patients. Tumor staging were classified according to NCCN (2010) guidelines as follow: 72 patients were pancreatic ductal adenocarcinoma, 15 patients were at stage I, 55 at stage II, 0 at stage III, and 2 at stage IV.

### Immunohistochemical analysis

Each tissue was fixed in formalin and embedded in paraffin in accordance with the standard procedures. Paraffin tissues were cut into 4 μm in thickness, then deparaffinized and rehydrated. Antigen retrieval was obtained by heating sections in a 10 μmol/l citrate buffer solution (pH =6.0) in a microwave for 10minutes. After that, sections were soaked in 3% hydrogen peroxide and absolute methanol to block the endogenous peroxidase activity. The 10% goat serum albumin was used in the sections for 20 minutes to block non-specific reaction. The sections were incubated overnight at 4°C with a primary antibody against human CIP2A protein (rabbit polyclonal antibody, Abcam Corporation, USA) at the dilution of 1:200. Sections were incubated with the secondary antibody for 1h at room temperature. Then sections were incubated with streptavidin coupled to horseradish peroxidase for 1 h and developed using diaminobenzidine (DAB) as a chromogen. In the end, the sections were counterstained with Mayer's haematoxylin. Negative control slides were duplicate sections as above except that the primary antibody was omitted. The CIP2A positive lung cancer tissues were used as positive controls.

We used the following scoring method to evaluate the expression of CIP2A. Each section were graded based on the average percentage of positive cells and the staining intensity in five areas (per x100 field) at random. The intensity (S) was graded as follows :0 (no staining),+1 (weak),+2 (moderate) and +3 (strong). According to the mean percentage (P) of positive cells, score of percentage were classified as : 0,<5%;1,5%-24%; 2,25%-49%; 3,<50%-75%; and 4,>75%. The stained sections were analyzed by two independent pathologists without knowing the characteristics of patients. The final H-score were obtained by using the arithmetic :H-SCORE = ∑ (S × P). The classification was as follow:-, 0;+,1-3; ++,4-7;+++,8-12.

### Cell culture

The human pancreatic cancer line cell lines SW1990, SW1990 gemcitabine resistant(SW1990 GR), BXPC3, Panc-1, ASPC-1, CAPAN-1, miapaca-2 and CFPAC-1 were preserved in our laboratory and cultured as recommended by American type culture collection (ATCC). hTERT-HPNE (DT) cells were maintained in medium D(1 V Medium M3, 3 V glucose-free DMEM, 5%FBS, 5.5 mmol/L glucose, 10 ng/mL EGF, and 50 mg/mL gentamycin).

### Quantitative RT-PCR

Total RNAs were extracted from gemcitabine-resistant cells and parental cells using Trizol reagent (Invitrogen, Carlsbad, CA, USA) according to the manufacturer's instructions and were reverse transcribed into cDNAs using the Promega AMV reverse transcription system (Promega, Madison WI, USA). Quantitative RT-PCR was performed with SYBR Green master mix real-time core reagents on an ABI 7500 (Applied Biosystems) according to the manufacturer's instructions. Primers for quantitative RT-PCR were as follows:

CIP2A-F: 5′-CGGACACTTGCTAGTATGTTG-3′
CIP2A-R: 5′-ATGTTCTTCAGCCACAGACTC-3′
Akt-F: 5′-TCGCTTCTTTGCCGGTATCG-3′
Akt-R: 5′-AGTAGGAGAACTGGGGGAAG-3′
BCL2-F: 5′-TGTGTGTGGAGAGCGTCAAC-3′
BCL2-R: 5′-CCCAGACTCACATCACCAAG-3′
β-actin-F: 5′ -AGAAAATCTGGCACCACACC-3′.
β-actin-R: 5′ -TAGCACAGCCTGGATAGCAA-3′.

The expression of mRNAs was normalized to that of the reference gene β-actin. Relative quantification of mRNA within the samples was caculated using the 2^−ΔΔCt^ method.

### Plasmid construction and generation of stable transfected cells

Three human CIP2A pshRNA plasmids (sh1, sh2, and sh3) were designed against three different CIP2A targets and constructed in pshRNA-iTS1-puro vector (Genloci) for SW1990 and in pshRNA-iTS1-G418 vector (Genloci) for SW1990/R. A scrambled sequence was designed as a negative control. All plasmids were verified by sequencing (AB1370, Applied Biosystems). Lentivirus was transfected with those plasmids. SW1990 cells were infected with CIP2A knockdown lentivirus containing pshRNA-iTS1-G418-sh and SW1990/R cells were infected with pshRNA-iTS1-puro-sh lentivirus. Cells were tested for the CIP2A expression after infection with the lentivirus. Vector sh3 had a knockdown efficiency ≥80% and was used for further studies. The CIP2A shRNA sequences were as follows: sh1: 5′-CACCGTCAGTACAAAGCCGTGAAGTTCA AGAGACTTCACGGCTTTGTACTGACTTTTTG-3′

sh2:5′-CACCGCTTCACTGATCTTAAGTATTTCAAGAGAATACTTAAGATCAGTGAAGCTTTTTG-3′

sh3:5′-CACCGCTGTCTCAACTAGCAGTAGATCAAGAGTCTACTGCTAGTTGAGACAGCTTTTTG-3′

and sh-control, 5′-CCGGCCTAAGGTTAAGTCG CCCTCGCTCGAGC GAGGGCGACTTAACCTTAGGT TTTTG-3′

The pshRNA-iTS1-G418-sh4 stable infected cells were selected by G418 monoclonal screening, whereas pshRNA-iTS1-puro-sh3 stable infected cells were selected by puromycin monoclonal screening.

### Drug sensitivity assay

Aliquots of 2×10^3^SW1990 or SW1990/R cells or CIP2A knockdown cells were seeded in 96-well plates with appropriate growth medium at 200 μl per well. After a 12-h recovery period, triplicate wells were exposed to various concentrations of gemcitabine for 72 h. The effects on cell growth were examined by the MTT assay as described previously. The light absorbance was measured by a Microplate Reader (Multiscan MK3, Thermo Lab system, USA) at a wave length of 490 nm. The cell survival rate (SR) was calculated using the formula: SR = (mean absorbance of the test well/mean absorbance of the control) × 100%; the inhibition rate (IR) was calculated using the formula: IR =100% - SR.

### Cell death and apoptosis assays

SW1990 and SW1990/R cells were exposed to gemcitabine-containing culture media at various concentrations of gemcitabine. After 24 h, the cells were harvested and stained with Annexin-V and PI using the Vybrant Apoptosis Assay Kit (Molecular Probes) per the manufacturer's protocol. Briefly, all cells were harvested by trypsinisation and washed twice with cold PBS. The pellets were resuspended in 100 μL 1× Annexin binding buffer and 5 μl fluorescein isothiocyanate(FITC)–Annexin-V (component A). A 1-μL working solution of PI at 100 μg/mL was added to each 100 μL of cell suspension. The cells were incubated on ice for 1 hr, washed again with cold PBS and re-suspended in 300 μL 1×Annexin-binding buffer. The stained cells were immediately analyzed by flow cytometry using FACScan Flow Cytometer (BD Biosciences, San Jose, CA)

### Western blot

The concentration of total protein extracted from parental and gemcitabine-resistant cells was determined with a BCA Protein Assay Kit (Pierce, USA). Equal amounts of protein were separated by 10% SDS-PAGE and electrophoretically transferred to PVDF membranes (Millipore, Bedford, USA) using a mini trans-blot (Bio-Rad laboratories, Hercules, CA, USA). Rat anti-human CIP2A, p- AKT(Millipore, Bedford, USA), and BCL2 (Abcam, MA, USA) were used to detect the expression of these proteins. β-actin (Santa Cruz Biotechnology, Inc., Santa Cruz, CA, USA) was used as an internal control. Electrochemiluminescence was performed according to the manufacturer's instructions and read with a Chemi lmager 5500 imaging system (Alpha Innotech Co, San Leandro, CA, USA).

## Abbreviations

CIP2A: Cancerous inhibitor of protein phosphatase 2A
PDAC: Pancreatic ductal adenocarcinoma
°C: Celsius
DAB: Diaminobenzidine
PBS: Phosphate buffered saline
HR: Hazard ratio
